# Murine Models of Lysosomal Storage Diseases Exhibit Differences in Brain Protein Aggregation and Neuroinflammation

**DOI:** 10.3390/biomedicines9050446

**Published:** 2021-04-21

**Authors:** Jennifer Clarke, Can Kayatekin, Catherine Viel, Lamya Shihabuddin, Sergio Pablo Sardi

**Affiliations:** Rare and Neurologic Diseases Research Therapeutic Area, Sanofi, 49 New York Ave., Framingham, MA 01701, USA; jennifer.matthews@sanofi.com (J.C.); can.kayatekin@sanofi.com (C.K.); catherine.viel@sanofi.com (C.V.); lshihabuddin@gmail.com (L.S.)

**Keywords:** lysosomal diseases, synuclein, tau, neuroinflammation, mouse models of disease

## Abstract

Genetic, epidemiological and experimental evidence implicate lysosomal dysfunction in Parkinson’s disease (PD) and related synucleinopathies. Investigate several mouse models of lysosomal storage diseases (LSDs) and evaluate pathologies reminiscent of synucleinopathies. We obtained brain tissue from symptomatic mouse models of Gaucher, Fabry, Sandhoff, Niemann–Pick A (NPA), Hurler, Pompe and Niemann–Pick C (NPC) diseases and assessed for the presence of Lewy body-like pathology (proteinase K-resistant α-synuclein and tau aggregates) and neuroinflammation (microglial Iba1 and astrocytic GFAP) by immunofluorescence. All seven LSD models exhibited evidence of proteinopathy and/or inflammation in the central nervous system (CNS). However, these phenotypes were divergent. Gaucher and Fabry mouse models displayed proteinase K-resistant α-synuclein and tau aggregates but no neuroinflammation; whereas Sandhoff, NPA and NPC showed marked neuroinflammation and no overt proteinopathy. Pompe disease animals uniquely displayed widespread distribution of tau aggregates accompanied by moderate microglial activation. Hurler mice also demonstrated proteinopathy and microglial activation. The present study demonstrated additional links between LSDs and pathogenic phenotypes that are hallmarks of synucleinopathies. The data suggest that lysosomal dysregulation can contribute to brain region-specific protein aggregation and induce widespread neuroinflammation in the brain. However, only a few LSD models examined exhibited phenotypes consistent with synucleinopathies. While no model can recapitulate the complexity of PD, they can enable the study of specific pathways and mechanisms contributing to disease pathophysiology. The present study provides evidence that there are existing, previously unutilized mouse models that can be employed to study pathogenic mechanisms and gain insights into potential PD subtypes, helping to determine if they are amenable to pathway-specific therapeutic interventions.

## 1. Introduction

Parkinson’s disease (PD) is the second most common neurodegenerative disorder, affecting approximately 1% of the population over 60 years of age. The initial description of PD consisted of mainly motor symptoms due to the degeneration of the nigrostriatal pathway. This definition has expanded in the last decade to include a variety of non-motor symptoms and aberrations in additional neural systems [1,2,3].

The accumulation of aggregated α-synuclein in neurons is the pathological hallmark of PD and related synucleinopathies [2]. The elucidation of the underlying molecular events leading to α-synuclein misfolding and aggregation is a field of active investigation. One prominent hypothesis is that lysosomal dysfunction in particularly susceptible neurons results in aberrant processing of proteins such as α-synuclein, leading to its abnormal aggregation. Substantial genetic and experimental evidence highlights a number of interrelated pathways that conspire to reduce the lysosomal hydrolytic activity, thus promoting aberrant misfolding of α-synuclein and associated proteins [4,5,6,7,8].

Mutations in the glucocerebrosidase gene (*GBA*) provided the first genetic evidence directly linking PD to lysosomal dysfunction. Homozygous and compound heterozygous *GBA* mutations cause Gaucher disease, an autosomal recessive lysosomal storage disorder (LSD), characterized by low lysosomal glucocerebrosidase activity and subsequent accumulation of lipid substrates [9]. Increased risk for PD in heterozygous carriers of *GBA* loss-of-function alleles was first recognized in families of individuals with Gaucher disease, also at an increased risk for the development of PD, [10,11] and confirmed by large, case-controlled genetic studies [12]. It is now well recognized that *GBA* variants increase the risk for PD and alter its clinical manifestations, causing earlier onset, increased risk of cognitive impairment, and overall accelerated disease progression [13,14].

Diminished lysosomal degradation ability has been proposed as a main contributor to PD pathogenesis [7,15]. The presence of proteinaceous aggregates including α-synuclein suggests that defective protein processing may be implicated in disease pathogenesis. Gaucher disease is one of more than 50 disorders related to loss of lysosomal enzyme activity. Loss of function mutations in several other lysosomal enzyme genes have also been identified as risk factors for developing PD and other synucleinopathies [6,16,17,18]. While not all genetic risk factors for PD cause lysosomal storage disorders (LSDs), or vice versa, the breadth of the shared genetic factors underlying PD and LSDs strengthen the connection between lysosomal dysfunction and synucleinopathies, and suggest that additional LSDs might be associated with PD.

Animal models of LSDs carrying knock-in mutations in the murine locus provide translatable platforms for understanding disease pathogenesis and enable the development of effective treatments for these devastating diseases [19,20,21]. These animals have also been used to model various features of synucleinopathies and the effects of potential therapeutic interventions currently undergoing clinical testing [22]. Loss of glucocerebrosidase activity in the *Gba^D409V/D409V^* Gaucher disease mouse model is associated with progressive, neuronal, proteinase K-resistant alpha-synuclein accumulation, progressive glucosylsphingosine accumulation in the central nervous system (CNS) and cognitive decline. These mice also accumulate ubiquitin and tau in these inclusions, similar to Lewy body pathology observed in synucleinopathies [23,24]. Importantly, these murine phenotypes occur in the context of normal endogenous protein levels and do not require exogenous overexpression of α-synuclein or other proteins. In addition, heterozygous *Gba^WT/D409V^* mice also harbor these aggregates, but to a lesser degree, suggesting a gene-dosage effect similar to the human condition, where synucleinopathy risk is higher in homozygous *GBA* mutation carriers than for heterozygotes [23].

Preclinical studies linking synucleinopathies and *GBA* mutations served as a proof of principle for investigating its association with other LSDs. In the present study, we evaluated the presence of aggregated protein pathology and inflammation in the CNS of several mouse models of LSDs, namely Gaucher, Fabry, Sandhoff, Niemann–Pick A (NPA), Hurler, Pompe and Niemann–Pick C (NPC, Table 1). All seven LSD models exhibited evidence of proteinopathy and/or inflammation in the CNS. Notably, these signals were divergent with models presenting various degrees and associations of aberrant protein accumulation and neuroinflammatory signals. These results suggest that substrate accumulation and the ensuing lysosomal dysfunction might differentially influence the progression of neuropathological changes in PD and LSDs. The present results suggest a greater number of lysosomal dysfunctions than previously appreciated can trigger pathologies associated with synucleinopathies, thereby provide additional models for studying potential therapeutic interventions.

## 2. Materials and Methods

Animals: All procedures were approved by the Institutional Animal Care and Use Committee at Sanofi and animals were housed in AAALAC accredited facility in compliance with all institutional, state and federal guidelines. Animals were group housed in polycarbonate rectangular cages under 12-h light:dark cycles, monitored daily and provided with food and water ad libitum. Symptomatic lysosomal storage disease model mice were obtained at various months of age. Here, symptomatic was defined by evidence of substrate storage accumulation (Table 1). Most of the models evaluated demonstrate substrate accumulation by 2 months of age, with variable lifespans following onset (Table 1). Tissue was collected between onset of storage and predicted end of life. For each genotype, 5 mice were analyzed, with the exception of the WT and Gaucher models, where one mouse was selected per genotype as negative and positive controls. Both control mice were 12 months of age, and historical data was incorporated into Table 2 and Table 3 [23,24].

For euthanasia, mice were injected intraperitoneally with sodium pentobarbital (Virbac AH, Inc., Fort Worth, TX, USA) and transcardially perfused with cold PBS (Mediatech, Inc., a Corning Subsidiary, Manassas, VA, USA) for 2 min at a rate of 18 mL/min. Mice were given 500 units of heparin (Pfizer, Inc., New York, NY, USA) prior to perfusion. Brains were harvested for histology and post-fixed in 10% neutral buffered formalin (VWR, Radnor, PA, USA) for 48 h prior to sucrose cryopreservation, OCT embedding and cryostat sectioning at a thickness of 20 microns.

Immunohistochemistry: All cryosections were blocked with PBS containing 10% normal Donkey Serum (Jackson Immunoresearch, West Grove, PA, USA)and 0.3% Triton X-100 (Sigma, St. Louis, MO, USA) and stained with rabbit anti-glial fibrillary acidic protein (GFAP, Dako, Carpinteria, CA, USA, 1:2500), mouse antineuronal nuclei (NeuN, Millipore, Burlington, MA, USA, 1:100), neurofilament high (NFH, Millipore, Burlington, MA, USA, 1:1000), rabbit anti-Iba1 (Wako, Richmond, VA, USA, 1:500) and mouse antimicrotubule-associated protein 2 (MAP2, Sigma. St. Louis, MO, USA, 1:250) in conjunction with appropriate Alexa Fluor conjugated secondary antibodies (Thermo Fisher, Waltham, MA, USA, 488 and 555, 3–4 uL/mL). For proteinase K-resistant α-synuclein, sections were pretreated with proteinase K (Dako, Carpinteria, CA, USA, 1:4 dilution) for 7 min, followed by block as described above, and stained with rabbit anti-α-synuclein (Sigma, St. Louis, MO, USA, S3062, 1:300). A cyanine 3 tyramide amplification kit was used to detect α-synuclein (PerkinElmer, Waltham, MA, USA). All slides were coverslipped with Aqua Poly/Mount (Polysciences, Warrington, PA, USA).

Scoring and imaging: The stained slides were analyzed using a Nikon Eclipse E800 upright microscope equipped with 488 and 555 filter cubes. The evaluator was blinded to the genotype of the mice and visually assessed phenotypes for protein aggregation and neuroinflammation, producing a phenotype score according to prespecified criteria. For alpha-synuclein and tau immunostaining, the following designations were applied: + = mild (more than 2 aggregate foci within the specified brain region), ++ = moderate (5 or more foci within the specified brain region) and +++ = severe (10 or more foci within the specified brain region). For Iba1, microglial activation scores were designated as follows: + = mild activation (increased cellular size within the specified brain region), ++ = moderate (increase in size and number within the specified brain region) and +++ = severe (large cells and extensive infiltration within the specified brain region). For GFAP, neuroinflammation was assessed as either increased (+), decreased (-) or no change (nc) compared to wild-type within the specified brain region.

For all assessments, hippocampus, cerebellum, cortex and brainstem were evaluated with a thorough visual scan of the entire immunostained tissue sections. For each brain region surveyed, an overall score was assigned to reflect the observed degree of phenotype severity. *n* = 5 animals surveyed per mouse model. For mice with multiple ages of sacrifice, the scoring represented a pooled assessment of the age groups. Only representative images of the hippocampus (for protein aggregation and neuroinflammation) and the cerebellum (for neuroinflammation), the regions that had the most prominent differences across all the animal models, were recorded.

## 3. Results

### 3.1. Mouse Models of Gaucher, Fabry and Hurler Disease Exhibit Proteinase K-Resistant α-Synuclein Pathology in the Brain

**Figure 1:**
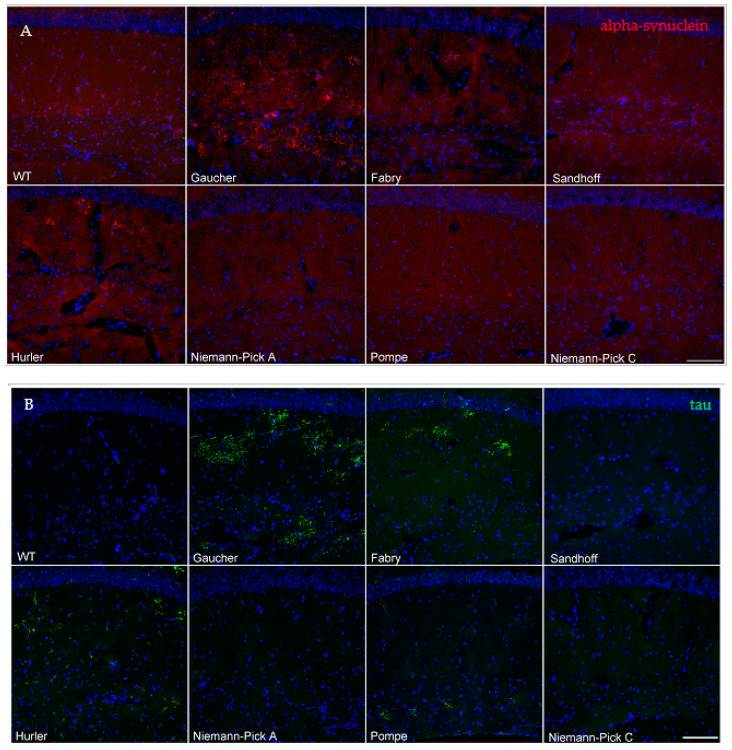
Gaucher, Fabry, Pompe and Hurler disease model mice exhibit aberrant proteinase K-resistant α-synuclein and/or tau immunoreactivity. The representative images show proteinase K-resistant α-synuclein immunoreactivity (red) in the hippocampi of wild-type or LSD mice (**A**, see Table 1 for details). The WT animal was 12 months old at sacrifice. Alpha-synuclein pathology was undetected in wild-type, Sandhoff, Niemann–Pick A, Pompe and Niemann–Pick C mice. Representative images from hippocampal tau immunoreactivity (green) revealed aberrant tau accumulation in the Gaucher, Fabry, Hurler and Pompe mice (**B**). Abnormal tau foci were nearly absent in the Sandhoff, Niemann–Pick A and Niemann–Pick C mice. Nuclei are shown in blue with DAPI counterstaining (scale bar, 100 μm).

Tissues from symptomatic Fabry, Sandhoff, Niemann–Pick A, Hurler, Pompe and Niemann–Pick C mouse models were collected and stained alongside Gaucher disease and wild-type mouse controls. The age at tissue collection varied according to each model’s disease progression and survival (Table 1). As we have previously described, the hippocampi of twelve-month-old homozygous *Gba^D409V/D409V^* mice displayed prominent neuronal proteinase K-resistant α-synuclein aggregates that were visualized by immunofluorescence [23]. We used a single *Gba^D409V/D409V^* animal as a positive control for these measurements. Similar aggregates were observed in the hippocampi of a subset of the Fabry and Hurler disease model mice, aged 9 and 7 months, respectively. These aggregates tended to accumulate within the pyramidal and molecular layers, where there are various inputs and outputs contained within the hippocampus and with other regions (Figure 1A). These aberrant proteinase K-resistant α-synuclein aggregates were also observed in the cerebella of the same animals (Table 2) and were nearly absent in the 12-month-old wild type control (Figure 1A). Of note, Gaucher disease model mice displayed additional proteinase K-resistant α-synuclein aggregates in the cortico-amygdala area as previously reported [23]. However, aggregates in this brain region were undetected in the Fabry and Hurler disease model mice (Table 2). No proteinase K-resistant α-synuclein aggregates were observed in any brain regions of Niemann–Pick disease (A or C), Pompe or Sandhoff disease mice (Figure 1A, Table 2). These results suggest that lysosomal dysfunction alone was insufficient for the development of α-synuclein proteinopathy, and that other underlying biochemical and cellular factors might contribute to the observed pathology.

### 3.2. Mouse Models of Gaucher, Fabry, Hurler and Pompe Disease Display a Diverse Distribution of Aberrant Protein Aggregation, Including Tau

Tau and α-synuclein are partially unfolded proteins that can form toxic oligomers and abnormal intracellular aggregates under pathological conditions. α-synuclein pathology frequently co-occurs with tau and other microtubule protein inclusions in the brains of mouse models of disease and synucleinopathy patients [24,25]. We therefore sought to characterize the presence of aggregated tau and MAP2 (2a + 2b) in the brains of LSD animals and evaluate their potential co-occurrence with α-synuclein pathology.

Gaucher, Fabry and Hurler mice demonstrated tau and MAP2 (2a + 2b) inclusions that colocalized with α-synuclein protein deposits (Table 2, Supplementary Figure S1). Interestingly, all the Fabry and Hurler disease model mice exhibited tau inclusions in the hippocampi and cerebella, even in animals where proteinase K-resistant α-synuclein aggregates were undetected (Figure 1B). This pathology extended beyond these regions in some of the Hurler disease model mice, where tau aggregation was also present in the brainstem (Table 2).

We were unable to detect abnormal MAP2 (2a + 2b) or tau inclusions in Sandhoff or Niemann–Pick (A or C) disease animals (Figure 1B, Table 2, Supplementary Figure S1), coinciding with the lack of abnormal α-synuclein aggregates in these models. Moreover, no aberrant protein pathology was observed in the nigrostriatal pathway regions of any of the models studied, similar to the α-synuclein aggregation results.

Lastly, Pompe disease mouse brains showed a remarkable pathological pattern. Despite the young age of 3–4 months, many animals exhibited aberrant tau pathology (Figure 1B) in all brain areas surveyed (Table 2). Like the Hurler disease model mice, Pompe disease mice exhibited prevalent brainstem tau aggregation. However, we were unable to detect aberrant α-synuclein (Figure 1A) or MAP2 (2a + 2b) (Supplementary Figure S1) staining in these mice, suggesting that glycogen accumulation might have a specific effect on the tau misfolding or simply that these methods were not sensitive enough to detect α-synuclein pathology in this model or at its particular disease stage.

### 3.3. Neuroinflammatory Profiles of Lysosomal Storage Disease Model Mice Differ in Severity and Distribution and Do Not Correlate with the Presence of Abnormal Protein Aggregates

LSDs have a heterogenous presentation and most patients develop CNS manifestations, especially those with significantly diminished enzymatic activity [26]. Hence, neuroinflammatory responses are a common feature of advanced lysosomal diseases [27]. To gain insights into the distribution of inflammatory signals and the potential association with areas of marked proteinopathy, we evaluated Iba1 and GFAP immunoreactivity by immunofluorescence. Microglial infiltration and activation were assessed using Iba1, a microglial calcium binding marker. Astrogliosis was evaluated via an established astroglia marker, GFAP (glial fibrillary acidic protein).

**Figure 2:**
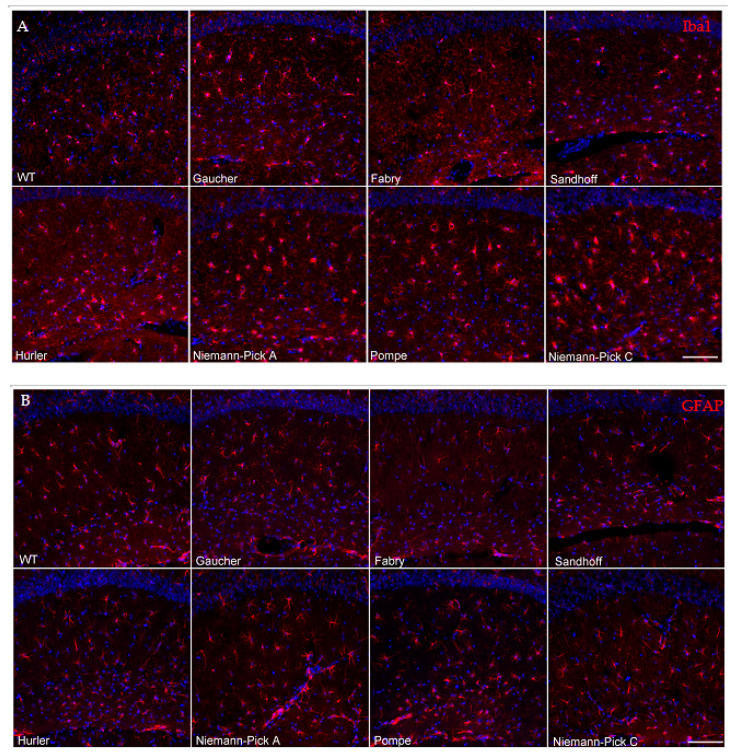
Neuroinflammatory profiles of lysosomal storage disease model mice hippocampi differ in severity and do not correlate with the presence of abnormal protein aggregates. Hippocampal Iba1 immunoreactivity (**A**) revealed mild to moderate microglial activation in the Sandhoff, Niemann–Pick A, Hurler and Pompe models, as evidenced by overt enlargement of cell bodies. The Niemann–Pick C mice displayed the most severe microglial activation of the disease models evaluated. No activation was observed in the Gaucher and Fabry mice. GFAP immunoreactivity (**B**) revealed only slight astroglia increases in the Pompe model, with no overt changes in astrocytosis in Gaucher, Fabry, Sandhoff, Niemann–Pick A, Hurler and Niemann–Pick C disease model mice. Nuclei are shown in blue with DAPI counterstaining (scale bar 100 μm).

Gaucher disease model mouse brains showed no signs of microglial activation via Iba1 immunostaining in any regions assessed, in agreement with our previous report showing no evidence of neuroinflammation in aged *Gba^D409V/D409V^* mouse hippocampus compared to wild-type controls. [23]. Similarly, brains from Fabry disease model mice showed no evidence of abnormal Iba1 signaling compared to wild-type controls (Figure 2A, Table 3).

**Figure 3:**
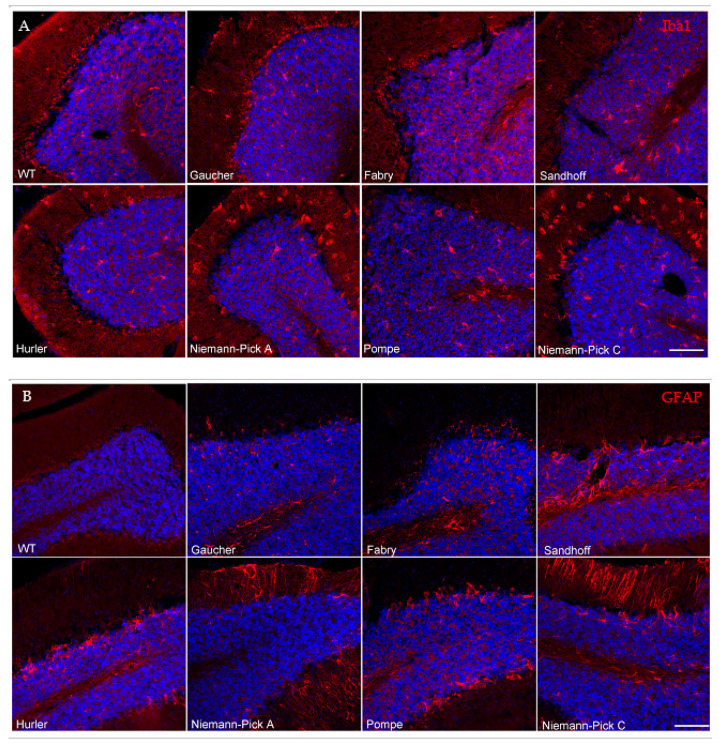
Cerebellar neuroinflammatory profiles of lysosomal storage disease model mice differ in severity and distribution. Representative images taken of Iba-1 immunoreactivity (**A**, red) and GFAP immunoreactivity (**B**, red) in the cerebella of seven LSD models. Sandhoff, Niemann–Pick A, Hurler, Pompe and Niemann–Pick C disease models mice all exhibited microglial cell activation in various cerebellar regions. Sandhoff and Pompe disease models exhibited microglial activation primarily in the granular and white matter cerebellar regions. Both Niemann–Pick disease models displayed widespread microglial activation in all layers. Hurler mice displayed mild to moderate microglial cell activation, primarily in the granular layer. GFAP immunoreactivity was prominent in Sandhoff, Niemann Pick A and C and Pompe disease models, with little to no changes observed in the other models. Nuclei are shown in blue with DAPI counterstaining (scale bar 100 μm).

Conversely, all other models evaluated in the present study showed some degree of microglial activation by Iba1 staining in every region interrogated. The Sandhoff disease mouse model demonstrated mild microglial activation in all assessed regions (Figure 2A, Table 3). As expected, the Niemann–Pick A disease model mice exhibited microglial infiltration and activation in the cerebellum (Table 3, Figure 3A), a site of major neuropathology in this model, with progressive lysosomal storage of sphingomyelin, degeneration of the Purkinje cell neurons and consequent motor behavioral abnormalities [28]. Notably, Niemann–Pick A neuroinflammation was distributed beyond the known regions of neurodegeneration and was widespread, much like in the Pompe disease model (Table 3). The spread of neuroinflammation appears to mirror the widespread substrate accumulation reported in these models [28,29]. Hurler disease model mice exhibited mild to moderate microgliosis in all regions evaluated. Niemann–Pick C disease model mice displayed severe microglial cell activation and infiltration in the hippocampus and cerebellum (Figure 2A and Figure 3A, respectively), in addition to the other regions evaluated (Table 3), correlating with extensive substrate accumulation described in this model [30,31]. Together, these results suggest that lysosomal substrate accumulation, rather than α-synuclein or tau protein aggregates, associates with microglial cell activation in these LSD models.

Brains from these various LSD models also displayed dissimilarities in the distribution and extent of astrogliosis, determined via GFAP immunofluorescence (Table 3). Gaucher, Fabry and Hurler disease model mice were free of any overt astrogliosis in all regions evaluated (Figure 2B and Figure 3B, Table 3). All Sandhoff disease model mice displayed GFAP-positive immunoreactivity in the cerebellum (Table 3, Figure 3B), consistent with previously reported GM2 ganglioside storage in this region and the consequent motor phenotype observed in these mice [32]. Additionally, a subset of the Sandhoff disease mice (60%), presented with astrogliosis in the cortex (Table 3). Niemann–Pick A disease model mice displayed astrogliosis in the cerebellum, the major site of neurodegeneration in this model [28]. Notably, a subset of Niemann–Pick C brains (60%) displayed increased GFAP immunoreactivity in the cerebellum (Figure 3B, Table 3), contrasting with the widespread microglial activation. Pompe brains showed a trend towards GFAP inflammation in the hippocampus and cerebellum (Figure 2B and Figure 3B, Table 3).

Neuroinflammation, embodied by Iba1 or GFAP staining, was observed in most of the models evaluated, with the exception of Gaucher and Fabry disease mouse models (Table 3). Remarkably, the pattern of astrogliosis did not necessarily correlate with that of microgliosis, suggesting a cell-specific response to the lysosomal substrate accumulation. Congruently, neuroinflammation was not necessarily associated with the presence of aberrant neuronal protein aggregates (Table 4).

## 4. Discussion

A common feature of advanced PD and related neurodegenerative conditions is the deposition of amyloid-like protein aggregates in the CNS. Accumulation of α-synuclein and other proteinaceous aggregates in postmitotic neurons is hypothesized to interfere with critical cellular functions leading to increased vulnerability and neuronal death [33,34]. Substantial evidence highlights the importance of lysosomal mechanisms in the abnormal accumulation of aggregated α-synuclein [4] and PD susceptibility [6]. The initial discovery of increased frequency of *GBA* mutations in PD led to the identification of multiple LSD gene mutations associated with PD and other synucleinopathies [8,16,35].

Much like PD and related synucleinopathies, LSDs patients exhibit a wide range of clinical presentations [36]. However, the intrinsic mechanisms of most LSDs are well defined, contrasting with the complex etiologies leading to PD. LSDs are typically caused by autosomal recessive gene mutations, resulting in a specific lysosomal enzyme deficiency and subsequent specific substrate accumulation, which can be pharmacologically normalized by correcting the metabolic defect [37]. The availability of translatable LSD animal models has proven critical for the successful development of effective therapeutics [19,20,21,37]. In contrast, no single mouse model can recapitulate the complex features of PD, and the use of diverse and complementary models would be necessary to deconstruct this complexity and elucidate the potential contribution of lysosomal dysfunction on disease pathophysiology [38,39].

In this study, we evaluated two hallmarks of PD, namely neuroinflammation and protein aggregation in a number of LSD mouse models. All animal models were homozygous knockout or knockin mutants for an LSD causal gene and tissues were collected after the onset of symptoms and/or storage pathology (Table 1). Importantly, these animals express only endogenous levels of murine α-synuclein and tau, analogous to the human condition and contrasting with models with overexpressed or exogenously applied fibrilized protein [38]. In addition, α-synuclein pathology was not observed in the substantia nigra or striatum of any of the models evaluated, in accordance with our previous report in *Gba^D409V/D409V^* mice [23]. However, α-synuclein proteinopathy was present in other brain regions implicated in synucleinopathies, such as the cortex and hippocampus [40,41]. The lack of overt proteinopathy in the mouse nigrostriatal region might be a reflection of key metabolic differences in the dopaminergic neurons of rodents and humans [42,43,44].

All disease animal models evaluated in the present study displayed evidence of protein aggregation and/or neuroinflammation (Table 4). While there was no obvious correlation between protein aggregation and neuroinflammation, as is observed in PD, more sophisticated techniques to evaluate neuroinflammation, such as panels of inflammatory markers beyond those applied here or morphological analyses of the cells, may yet reveal if such associations exist. Gaucher and Fabry disease models showed marked proteinopathy, which was not associated with neuroinflammatory signals. Conversely, Sandhoff, NPA and NPC disease mouse models exhibited marked neuroinflammatory pathology without any overt α-synuclein or tau proteinopathy. These results suggest that lysosomal dysfunction alone is not a unique driver of protein misfolding, but that specific pathways related to particular lysosomal functions and/or lysosomal substrate classes might play a larger role [45]. In addition, these results propose that neuroinflammation does not necessarily associate with neuronal aggregate formation. This divergence suggests that alternative or more complex pathogenic mechanisms might underlie the neuroinflammatory process, or that the pathological insult in these LSD models is relatively mild and therefore a more severe insult would be necessary to initiate the glial response [23].

The Pompe disease model mice presented notable features compared to the rest of the LSD models evaluated in the present study. Pompe disease is caused by mutations in *GAA*, the gene encoding the lysosomal enzyme acid alpha-glucosidase, resulting in decreased enzymatic activity and subsequent glycogen accumulation. Although Pompe has long been considered a purely metabolic muscle disorder, there is now increased recognition of CNS involvement as life expectancies of patients treated with non-brain-penetrant enzyme replacement therapy are extended [46]. The Pompe disease model mice demonstrated a unique and extensive distribution of tau aggregates, without evident α-synuclein inclusions, and with 100% of the animals displaying irregular tau immunostaining in the brainstem (Table 2). This model demonstrates glycogen accumulation in the brainstem [47]. The presence of atypical tau protein aggregation occurring independently of α-synuclein in a disease model where the accumulating substrate is glycogen, a polysaccharide (vs. a lipid), is intriguing and warrants further investigation [48].

The present study has several limitations that should be taken into consideration. Five of the seven models used are homozygous knockouts for critical lysosomal genes versus carrying a knockin point mutation rendering low residual enzymatic activity. Total loss of protein expression can result in different outcomes compared to the expression of mutant proteins. For instance, the burden of folding a mutant protein and other such effects cannot be recapitulated in a strict knockout. Additionally, the complete loss of enzyme activity is rarely found in human patients except in the most severe cases (Platt et al., 2018). Another limitation of animal models is the age at which these mice were evaluated. Due to the variable disease course, lifespan and disease severity in some of these mouse models, such as the NPA, Sandhoff and NPC mice, animals were sacrificed at a younger age than other models harboring less severe disease phenotypes. In order to represent a time when lysosomal dysfunction is having an impact on animal health, tissues were collected following the reported onset of lysosomal storage pathology and prior to the disease end stage. Thus, a lack of protein aggregation or neuroinflammation at a single, symptomatic timepoint cannot infer their absence at a later time. Lastly, the lack of observed pathology in a particular model does not preclude its presence, as undetected signals might be revealed with more sensitive methods.

PD is a complex disease with slow progression and multiple etiologies. Many animal models have been developed to understand PD pathogenesis and mimic its disease progression. While no model can replicate the complexity of the human condition, they can be suitable to study specific pathways and mechanisms and have proven critical to the advancement of successful therapeutics. As our understanding of the complexity of PD advances, the present study uncovers additional model systems to study pathogenic mechanisms and novel insights into potential disease subtypes amenable to specific therapeutic interventions.

## Figures and Tables

**Table 1:** Mouse models of metabolic disease.

Disease	Genotype	Deficient Enzyme	Accumulating Substrate(s)	Storage/Symptom Onset (Months)	Lifespan (Months)	Age @ Sacrifice (Months)	References
**Gaucher**	*Gba^D409V/D409V^*	Glucocerebrosidase	glucosylceramide, glucosylsphingosine	2	18–24	12	(Xu et al., 2003; Sardi et al., 2011)
**Fabry**	*Agal^-/-^*	α-galactosidase	Globotriaosylceramide	1–2	18–24	8–9	(Bangari et al., 2015)
**Sandhoff**	*HexB^-/-^*	β-hexosaminidase	Ganglioside GM2	3–4	5	2–3 & 4–5	(Sango et al., 1995; Cachon-Gonzalez et al., 2006)
**NPA**	*Smpd1^-/-^*	Acid Sphingomyelinase	Sphingomyelin, cholesterol	1–2	7	4–5	(Horinouchi et al., 1995)
**Hurler**	*Idua^W392X/W392X^*	α-L-iduronidase	Mucopolysaccharides	<1	10–20	4 & 7	(Wang et al., 2010)
**Pompe**	*Gaa^-/-^*	α-glucosidase	Glycogen	1–2	18–24	3–4	(Raben et al., 1998)
**NPC**	*Npc1^-/-^*	N/A-molecular transporter deficiency	Cholesterol	1–2	3	1 & 2	(Morris et al., 1982)

Abbreviations: NPA = Niemann Pick A, NPC = Niemann Pick C.

**Table 2:** Regional distribution of Lewy-body-like protein aggregates in brains of LSD model mice.

	α-Synuclein				Tau				MAP2(2a + 2b)			
	HC	CB	CX	BS	HC	CB	CX	BS	HC	CB	CX	BS
**Gaucher** *	100%	100%	100%	0	100%	100%	100%	0	100%	100%	100%	0
**Fabry**	40%	40%	0	0	100%	100%	0	0	40%	40%	0	0
**Sandhoff**	0	0	0	0	0	0	0	0	0	0	0	0
**NPA**	0	0	0	0	0	0	0	0	0	0	0	0
**Hurler**	20%	20%	0	0	100%	100%	0	60%	20%	20%	0	0
**Pompe**	0	0	0	0	60%	80%	40%	100%	0	0	0	0
**NPC**	0	0	0	0	0	0	0	0	0	0	0	0

Percent of mice exhibiting protein aggregates are shown. *n* = 5 per model. * Data from Gaucher model from historical data (Sardi et al., 2011; Sardi et al., 2013). See Methods for experimental details regarding staining and blinded semiquantitative assessment. Abbreviations: HC = hippocampus, CB = cerebellum, CX = cortex, BS = brainstem.

**Table 3:** Microglial activation and astrocytic GFAP expression in brains of LSD model mice.

	Iba1				GFAP			
	HC	CB	CX	BS	HC	CB	CX	BS
**Gaucher**	nc	nc	nc	nc	0	0	0	0
**Fabry**	nc	nc	nc	nc	0	0	0	0
**Sandhoff**	+	+	+	+	0	100	60	0
**NPA**	++	++	++	++	0	100	0	0
**Hurler**	+	+	+	+	0	0	0	0
**Pompe**	++	++	++	++	20	80	0	0
**NPC**	+++	+++	+++	+++	0	60	0	0

Iba1 staining was assessed and compared to wild-type controls. GFAP immunoreactivity is quantified as percent of mice exhibiting increased signal in a particular brain area (*n* = 5/group, except Gaucher mouse model which was compared to historical controls (Sardi et al., 2011; Sardi et al., 2013). See Methods for experimental details regarding staining and blinded semiquantitative assessment. Abbreviations: Iba1: nc = no change compared to wild-type controls, + = mild activation, ++ = moderate activation, +++ = severe activation. GFAP: numbers show percentage of mice evaluated, which demonstrated increased astrogliosis.

**Table 4:** Summary of detectable proteinopathy and neuroinflammatory markers in brains of LSD model mice.

Disease	Genotype	Proteinopathy (Y/N)	Neuroinflammation (Y/N)	
			Iba1	GFAP
**Gaucher**	*Gba^D409V/D409V^*	Y	N	N
**Fabry**	*Agal^-/-^*	Y	N	N
**Sandhoff**	*HexB^-/-^*	N	Y	Y
**NPA**	*Smpd1^-/-^*	N	Y	Y
**Hurler**	*Idua^W392X/W392X^*	Y	Y	N
**Pompe**	*Gaa^-/-^*	Y	Y	Y
**NPC**	*Npc1^-/-^*	N	Y	Y

## Data Availability

The data that support the findings of this study are available from the corresponding author, upon reasonable request.

## Supplementary Materials

The following are available online at https://www.mdpi.com/article/10.3390/biomedicines9050446/s1, Figure S1: Hippocampal α-synuclein and MAP2(2a + 2b) Immunofluorescence, Table S1: Aggregate Stains Tau, a-SYN, MAP2 (see supplementary scoring data in excel workbook).
